#  Tumor suppressor genes in familial adenomatous polyposis

**Published:** 2017

**Authors:** Nahal Eshghifar, Naser Farrokhi, Tahereh Naji, Mohammadreza Zali

**Affiliations:** 1 *Department of Molecular and Cellular Sciences, Faculty of Advanced Sciences & Technology, Pharmaceutical Science Branch, Islamic Azad University, Tehran, Iran *; 2 *Department of Plant Biology & Biotechnology, Faculty of Biosciences & Biotechnology, Shahid Beheshti University, Tehran, Iran*; 3 *Gastroenterology and Liver Diseases Research Center, Research Institute for Gastroenterology and Liver Diseases, Shahid Beheshti University of Medical Sciences, Tehran, Iran*

**Keywords:** Familial adenomatous polyposis (FAP), Tumor suppressor genes (TSGs), Adenomatous polyposis coli *(APC)*

## Abstract

Colorectal cancer (CRC) is mostly due to a series of genetic alterations that are being greatly under the influence of the environmental factors. These changes, mutational or epigenetic modifications at transcriptional forefront and/or post-transcriptional effects via miRNAs, include inactivation and the conversion of proto-oncogene to oncogenes, and/or inactivation of tumor suppressor genes (TSG). Here, a thorough review was carried out on the role of TSGs with the focus on the *APC* as the master regulator, mutated genes and mal-/dysfunctional pathways that lead to one type of hereditary form of the CRC; namely familial adenomatous polyposis (FAP). This review provides a venue towards defining candidate genes that can be used as new PCR-based markers for early diagnosis of FAP. In addition to diagnosis, defining the modes of genetic alterations will open door towards genome editing to either suppress the disease or reduce its progression during the course of action.

## Introduction

Colorectal cancer (CRC) with almost one million new incidences each year is classified amongst aggressive malignancies. Among people with CRC, about 10% have a first-degree affected relative(s) with the disease (1), (2) and more than half of the gastrointestinal tract cancers are linked to diet (3), (4). CRC appears in many shapes and forms (5) and here familial adenomatous polyposis (FAP) that accounts for about 1% of CRC cases is going to be reviewed due to its general occurrence in earlier stages of life. 

**Figure 1:**
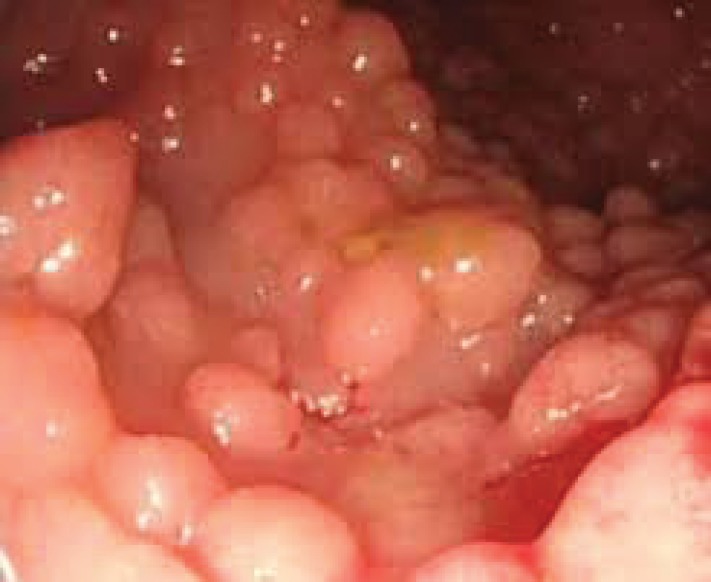
Endoscopic image of sigmoid colon of familial adenomatous polyposis (FAP)’ patient

Common FAP, Gardner syndrome, desmoids and attenuated FAP are all different forms of FAP (5-9). As the name suggests, numerous polyps and growth of the inner lining, are being formed in the colon and rectum (Figure 1). Most polyps are benign; however some polyps may advance to cancer (9-11). Similar to other cancers, FAP is the result of a series of genetic changes, including activation of oncogenes or inactivation of tumor suppressor genes (TSGs). Varieties of gene mutations and epigenetic variations in oncogenes and TSGs in addition to chromosomal changes may lead to FAP (10, 11). Mutations in TSGs lead to cell proliferation, avoiding programmed cell death. Consequently, in the absence of tumor suppressor proteins, cells, so-called “no brakes” begins their stunted growth. Chromosomal deletions bearing in* APC*, *DCC* and *P53* (10, 12, 13) have also corroborated with inactivation of TSGs (10, 12-15). 

Identification of new TSGs and their characterization remains an active area of cancer studies (16). In general TSGs, a suite of usually co-expressed genes and most often co-localized proteins, happened to have particular defined roles in certain cellular pathways (12, 17). The TSGs with more pronounced effects on FAP are presented in Table 1 (10,15, 16, 18-25).

 Genetic instability (GI), from a point mutation to chromosomal rearrangements and chromosomal instability (CIN), chromosome miss-segregation, the microsatellite instability (MSI) and CpG island methylator phenotype (CIMP) pathways all and all have a part in FAP formation either solely or in concert with each other (17, 26). Herein, TSGs and their relevant mutation in FAP and epigenetic alterations and the role of miRNAs were discussed.

**FAP TSGs and the corresponding mutations**

Genetic mutation shall be considered as the main factor that leads to the development of FAP. Studies of mutations are important from the perspective of molecular diagnostic, treatment, and more recently for genome editing purposes (27, 28). CRCs appear in two major forms, namely sporadic and hereditary (27-29). Mutations in TSGs contribute to the progression of m cancer by inactivating their inhibitory function. Mutations in TSGs are recessive at the level of an individual cell. Normally, a single mutation in one gene for malignant phenotype is not enough and more mutations required for carcinogenesis (10, 20, 28).

Among the early events in the FAP tumor progression pathway is the lack of the *APC* gene, a TSG, which appears to be consistent with its essential role in predisposing individuals with germ line *APC* mutations in colorectal tumors. Mutations within the *APC*, more than 300 mutations, have correlated with the onset of FAP. Once *APC* is mutated, tumor development is unavoidable (9, 10, 30). *APC* is being inherited in an autosomal dominant pattern, which implies one copy of the altered gene would be adequate for cancer formation. In addition, a mutation of *APC* must be followed by other mutations in some genes such as other TSGs to develop into cancer (31, 32). 

Mutant form of *APC* is found in sporadic and some types of hereditary CRC. Genetic transformation of mutant *APC* in FAP results in abnormal CRC cell proliferation, leading to the outgrowth of numerous adenomas in the colons of patients. Most of *APC* mutations are frame-shift mutations (33, 34) and population analyses have revealed the role of APC is indispensable in FAP formation (33-37). Based on many surveys (35, 38, 39), deletions at the codons 1309 (c.3927_3931delAAAGA) and 1061 (c.3183_3187delACAAA) were found to be the most frequent germ line mutations (35, 38, 39). The frequency of the first mutation differs from 0% in northern parts of Spain to 1.5% in an Israeli population and 8.4% for Australian FAP patients. The frequency of the second mutation varies from 16% in Italian, 7% in the Israeli population, 5% for Dutch, 0% in southwest of Spain, and 2.4% for Australian FAP patients (40, 41). More than 60% of *APC* mutations are observed within the mutation cluster region (MCR) between codons 1284-1580 or 1284–1464 (42, 43). Large rearrangements are more plausible to provide a classic FAP; while splicing mutations tend to result in attenuated FAP (AFAP) (44, 45). In a study on Greeks (44), two different cases of FAP families carrying two unusual *APC *mutations were observed: a splice site mutation on the fifth nucleotide of intron nine and a large genomic deletion in chromosome five. A diverse germ line–somatic *APC* mutation association was seen in FAP desmoids (44). Analysis of the *APC* in a Greek cohort (46) revealed 18 different germ line mutations (80%), distributed between exon 3 and codon 1503 of exon 15, while large genomic rearrangements were not identified (46). Additionally, a study in the Balearic Island indicated that the hotspot c.3183_3187delACAAA mutation has a significant effect on FAP (47). Few reports (44, 48) of mutations at position +5 in *APC* have shown that replacement of the +5 conserved nucleotide at the splice donor region of intron 9 within the *APC *provide frameshift and inefficient exon skipping (44, 48). Another study among the Iranian population (49) showed the detection rate of large fragment deletions in *APC* was 5.8% (2/34) and patients had deleted in the middle and at the end of exon 15. Comparison of clinical observations between patients without deletion and with the large deletion did not show any considerable difference in each variable, including signs of disease, age at diagnosis, and polyp type. It seems that exon 15 of *APC* gene is likely the hotspot zone in Iranian FAP patients (49). In a study of *APC* in 300 unrelated polish families with FAP, 97 types were recognized in 164 families; 80 small mutations and 17 large rearrangements, eight of which repeat rearrangements (40).

In fact, it seems that *APC* is part of many different signaling pathways that makes it a perfect target for mutation in FAP. Different types of mutation in the *APC* in FAP patients from different populations have presented in Table 2 (39, 40, 46, 47, 50-58). Altogether, it seems that the vast majority of *APC* mutations are small insertions/ deletions or point mutations. Splicing mutations rearrangements are less common inactivating events of the *APC* gene. M of 60% of *APC* mutations were described in exon 15. FAP patients usually harbor mutation at 5' (codons 78-167) and 3' (codons 1581-3843) regions of the *APC* (50, 59-61).

APC plays an important role in the Wnt signaling pathway. *APC* regulates the depreciation of β-Catenin**,** a transcription factor for proliferative genes, and its mutation leads to the accumulation of β-Catenin (62-64). The Wnt signaling pathway is remarkable in many cancers including FAP (65). 

In addition, mutation or deletion of *DCC* and *p53* and loss of cadherin leads to metastasis. The mutations within the *P53* lead to failure of apoptotic mechanisms and change in the regulation of cell proliferation, thereby causing genomic instability and malignant development. Clarification of candidate FAP biomarkers typically commences by examining the expression profiles of normal and cancerous tissues by performing high throughput gene expression profiling. Molecular markers in FAP such as mutations, genetic instability, and protein expressions have been shown to significantly influence the disease progression (15, 17, 59, 60).

**Genetic instability (GI) and chromosomal instability (CIN)**

**Figure 2:**
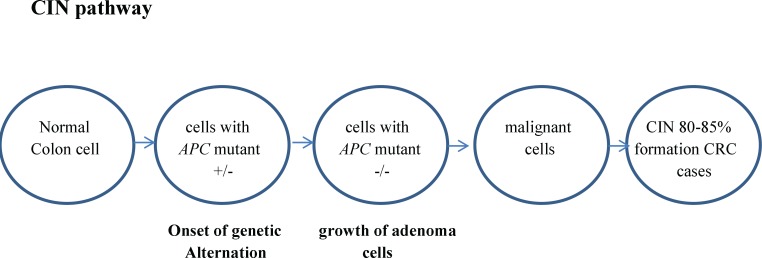
CIN in FAP

Genetic instability (GI) is associated with gene expression alterations (66). Analysis of transcripts as biomarker(s) allows predicting the process of cancer formation using GI as an indicator (21). Approximately 70–85% of CRCs develop via chromosome instability (CIN) pathway (Figure 2) (26). CIN is defined to be one of the driving forces that stimulate tumorgenesis in FAP (15, 17, 67, 68). Changes in chromosome number (aneuploidy) and high frequency loss of heterozygosity (LOH) are associated with CIN. It can result from defects in chromosomal dissociation, failure to respond properly to DNA damage, and telomere stability (69). Accumulations of numerical or structural chromosomal abnormalities lead to CIN pathway molecular aberrations (70-72). 

CIN and TSGs are somewhat associated; CIN results from a series of genetic alteration in *APC, DCC, P53 *and* SMAD4* (14, 17, 70). Multiple uncommon inactivating mutations of genes, the ones that their normal roles are to maintain CIN throughout replication, are evident. Aggregation of these mutations account for most CINs in FAP (26, 33).

**Wnt pathway**

**Figure 3:**
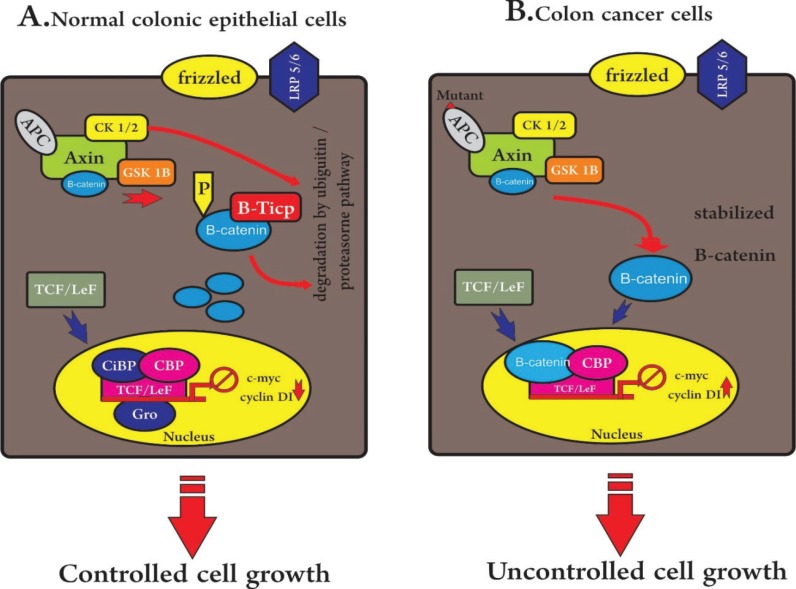
Wnt pathway in FAP

Wnts are glycoproteins where secretion is controlled particularly by a transmembrane protein, namely Wntless. Also the production of active Wnt needs a functional retromer that is a multiprotein complex involved in intracellular protein trafficking (73, 74). The Wnt signaling pathway has been reported of being altered in 93% of tumors, such as activating mutations of *CTNNB1* or biallelic inactivation of *APC* in 80% of FAP cases. The earliest genetic event in FAP is the activation of Wnt signaling via the genetic disruption of *APC* (32, 33). Activation of this pathway does not, by itself, lead to cancer, but it has a remarkable role in initiating the carcinogenic mechanism. Inherited mutations in *APC*, as in FAP, cause the improvement of non-invasive colonic adenomas (thousands of polyps) (19). Canonical Wnt signaling pathway has an essential role in the homeostasis of the colonic epithelium and its deregulation in CRC and more specifically in FAP*.* *APC* plays a central role suppressing the canonical Wnt signaling pathway that controls cell proliferation and differentiation in the intestine (65). Wnt signaling (Figure 3) (75) regulates growth, differentiation and apoptosis and is specifically relevant in maintaining tissue specific stem cell compartments. Wnt factor binds to cell surface Frizzled (FZD10) receptor (the Wnt receptor frizzled was overexpressed that activates disheveled (Dvl) in human through the activation of casein kinases. This inhibits the activity of a multiprotein complex consisting of β-Catenin, *Axin*, *APC* and glycogen synthase kinase (*GSK-3b*).  Normally this complex phosphorylates β-Catenin and thereby it is subjected to ubiquitination and proteosome-mediated degradation (74, 76).

In the absence of Wnt signaling, the β-Catenin accumulates within the cytoplasm and then moves to the nucleus to make complexes with T-cell factor (*TCF*). *TCF1* is a transcriptional target of the TCF4/β-Catenin complex and could provide a negative feedback mechanism for the complex (35, 64, 77-79). Subsequently, cMyc and other genes essential for unremitting cell cycle events are being up-regulated.

These genes are normally important for differentiation and stem cell renewal, but when inappropriately expressed at high levels, they can cause FAP (35, 80-82). The protein β-Catenin associated with other components is responsible for the activation of many genes, especially *cMyc*, responsible for inducing uninhibited cell cycle events. Increment of functional mutations in β-Catenin (*CTNNB1*) were diagnosed in half of the CRC tumors with intact *APC*, reflecting the significance of the Wnt pathway (17, 65). Altogether, it seems that, the most commonly mutated gene in Wnt pathway in FAP is the *APC*. Other genes associated with a Wnt pathway are listed in Table 3 (15, 64, 65, 77-85).

**The role of epigenetic modifications of TSGs in FAP**

In addition to genetic mutations, epigenetic modifications determine physiological and pathological cellular functions. Human cancers “such as FAP” harbor some of different heritable abnormalities in gene expression that are not resulting from mutation in any region of the genome, named epigenetic alterations (86). Epigenetics is described as heritable alterations in gene expression without DNA mutation and encompass three classifications: DNA methylation, histone and chromatin modification, and microRNA (miRNA) changes (69, 86-88).

Changes in DNA methylation patterns may lead to FAP. DNA hyper-methylation takes place at specific regulatory sites within the promoter regions or repetitive sequences. Methylation within the CpG islands of the TSG promoters can result in transcription blockage and therefore leading to cancer (89-91). Mechanisms related to gene inactivation, such as transcriptional silencing via promoter hyper-methylation, might play a role in loss of *APC *function in those tumors.

Methylation of *APC *has been represented in a subset of colorectal tumors (92, 93), but the exact role and density of this alteration, its functional consequences, and its tumor distribution need to be addressed in future. Subsequent to *APC *methylation, other *APC* pathway components can also be influenced (20). β*-*Catenin is a crucial downstream part of the *APC *pathway and *APC *promoter methylation may reduce its abundance (20).

The reports on methylation is rather contradictory in FAP; in a study by Zhang, et al (94) among FAP families, hyper-methylation was observed in tumor tissues once compared to normal tissues. In contrast, in a study by Segditsas, et al. (95) indifference in methylation behavior between the two tissue samples were noted (94, 95). So far, all studies insinuate that even though *APC *promoter methylation has taken place early throughout colon neoplastic development, it does not result in whole gene inactivation or act as a “2nd hit” and promoter-specific modifications of *APC *seldom leads to mutation-negative FAP (96).

Another type of epigenetic modification is histone and chromatin changes. Histones are alkaline proteins that function as scaffolds around which DNA winds in structures known as nucleosomes. Histones have protein tails that suppress gene transcription when specific methylation and acetylation arise. Post-transcriptional alterations, such as methylation, acetylation and phosphorylation are common events that adjust the biology of histones. Some histone proteins have already been determined to be overexpressed in different types of colon tumors (90, 97, 98). It has become obvious over the past two decades that epigenetic changes of the chromatin, especially the chromatin components in the promoter areas of TSGs, play remarkable roles in pathogenesis (90).

While there may be some understanding of how such genetic and epigenetic changes may affect the gene expression, and thereby tumor evolution, it is less clear how these mechanisms may have an effect on each other, and how these cumulative changes may coevolve and have an impact on gene expression inside the route of tumorgenesis (99).

Genomic imbalance and DNA methylation have been shown to disrupt normal gene expression and gene dosage, whereas disruptions of DNA methylation profiles may play a function in genomic instability especially at repeat-rich sequences.

Even though relatively less is known in regards to the patterns of particular histone alterations in FAP, selective histone alterations and resultant chromatin conformation have been shown to act in coordination with DNA methylation to adjust gene expression to mediate FAP pathogenesis. Furthermore, it is now clear that not only DNA methylation but also histone alterations are reversible tactics. Better comprehension of epigenetic regulation of gene expression within the context of pathogenesis has led to progress of epigenetic biomarkers for FAP diagnosis and epigenetic medications for FAP therapy (90, 99).

**MiRNA**

MiRNAs are short noncoding RNA sequences (19 to 25 nucleotides), which are involved in various biological processes such as cell death, differentiation and proliferation (100, 101). They induce gene suppression up to 30% (102). Modified miRNA expression has been reported in genes associated with FAP and specific miRNA patterns have described certain CRCs (86, 90).

MiRNAs are encoded within the genome, transcribed into forerunner transcripts, and undergo a series of strongly regulated processes leading to their incorporation in the RNA-inducing silencing complex (RISC). Thus, RISC directs the modulation of mRNAs translation by way of the binding of the miRNA to the 3' untranslated area of the target mRNA via a regional or whole sequence homology; accordingly, the translation of the miRNA would be down regulated or blocked, respectively (102-104).

Staphylococcal nuclease Tudor domain containing 1 (SND1) is a novel part of RISC in RNA-interference (RNAi) process. SND1 has been reported to be overexpressed in FAP and also in models of colon cancer. Overexpression of *SND1* can effect on activation of the Wnt-β-Catenin pathway via down regulation of the *APC*. Given the fact that *SND1* influences the onset of FAP via miRNA-mediated gene silencing, more researches are needed to further define how SND1 lead to FAP (105, 106).

The role of miRNA in regulation of APC expression is another topic of interest. So far, target sequences for miRNA as well as the location of three encoding genes were recognized. The *STAB1 *coding miR-135a-1 is located in 3q21 (first intron of the stabilin 1), while the *RMST* coding miR-135a-2 is in intron 5, coding the transcript related to rabdomyosarcoma (12q23) (107). Experimentally was shown that miR135a and miR135b interact with 3'UTR transcript of the *APC*, affecting the abundance of relative mRNA. Therefore, *APC *expression is being regulated and as a result the Wnt proliferation control signaling pathway. Increased levels of miR-135a and miR-135b caused the reduction in the *APC *mRNA abundance (10, 107). MiRNAs have been linked to many levels of cancer, from initiation and development of tumors in response to treatment, and development of new therapies. Lately, the role of altered miRNA expression in the Wnt pathway regulation and FAP advancement was also been evaluated. The studies inferred the decreased expression of miR-20b, miR-126, miR-143, and miR-145 as a primary event of FAP tumors (108, 109). It has been suggested that there is also a complex interaction amongst the various epigenetic changes, particularly after silencing of tumor suppressive miRNAs with the aid of promoter CpG methylation used to be suggested (110).

**Current Diagnostic Methodologies for FAP**

Multiplex ligation-dependent probe amplification (MLPA) (111) is commonly used for the concurrent evaluation of gene dosage and this technique is used to find exonic duplications and deletions with *APC*. MLPA is being considered as the first appropriate step in the analysis of a suspicious FAP cases. The relative analytical ease of MLPA versus the full gene scanning and the relative frequency of genomic rearrangements both justifies the use of MLPA . It needs to be stated that the present commercially MLPA kits have not yet been confirmed for diagnostic purposes (49, 112).

Cytogenetic analysis/ de novo and translocations disrupting *APC*, and inherited deletions that can be detectable cytogenetically were recognized in a small number of families/ individuals. Also, chromosome investigations could be considered, if no mutations were detected (6, 19, 112).

In cases where the mutation is evident, it is more likely that the individual develops FAP. Since *APC *mutations are nearly 100% penetrant, their onset most likely will develop into FAP. On the other hand, individuals with no familial mutation signifies that the development of FAP is highly unlikely. However, reports should point out that they continue to be at populace risk of sporadic CRC (34, 112). Prenatal testing and pre-implantation genetic diagnosis (PGD) is not often asking for FAP, but has been performed on a number of occasions (112).

Next generation sequencing (NGS) technology could be included as an appropriate diagnostic technique for the recognition of intragenic mutation testing of FAP. The multiplex NGS approach has the benefit of analyzing numerous genes in numerous samples, bearing the fact that technology is becoming more cost-effective as time goes by. Furthermore, with technology development the diagnostic turnaround times have been reduced immensely and continues to be further declined (113, 114, 115). The other main advantage of NGS is its capability to define the relevant genes that have remained elusive (116).

Pre-symptomatic testing (117), *APC *exon dosage analysis such as microsatellite analysis (112), exon-specific qPCR, linked SNP (112), cytogenetic FISH analysis (112, 118) and/or karyotyping array CGH or Southern analysis with cDNA probes (111, 112) would be the other techniques. However, these methodologies usually are not in common use (34, 112, 119).

## Discussion

Most FAP cases are resulting from germ line mutations in *APC*. Mutations are located mainly at the 5′ end, but extend all over the length of the *APC*. Most of which are small deletions/insertions or point mutations, and most result in N-terminus of protein fragments failing the protein in binding and degradation of β-Catenin and consequently cannot suppress Wnt/ β-Catenin pathway (34, 120).

Several studies have failed to correlate the lack of *APC* with activation of the Wnt pathway as determined by the presence of nuclear β-Catenin, especially in early adenomas (121-123). An absence of nuclear β-Catenin suggests involvement of other factors such as methylation. Taken together the studies declare that epigenetic changes cooperate with proliferative signals for initiation and development of colon tumors (124). Usually, most mutations are positioned in exon 15 of *APC* due to the fact it is the largest known exon (6557 bp long). Additionally, promoter hyper-methylation and loss of heterozygosity of *APC* has been reported in FAP and CRC (10, 17, 35, 125). Lack of *APC*, seems to induce improper DNA methylation. 

These modifications in DNA methylation, along with alterations in histone modifications, create a new perspective to explain failures in cell signaling and onset of FAP and other types of CRCs. Currently, some of causal mutations in FAP patients could not be diagnosed in *APC* and other polyposis-relevant genes such as *MUTYH *(34, 109, 126). Epigenetic cancer markers are just beginning to be understood, but are being inquired for use in early detection and prognosis, tumor classifications, following the effectiveness of chemotherapy, and providing targets of intervention. In précis, colon cancer genetics has organized genetic testing for hereditary syndromes and guides to therapy with obtained mutations, while epigenetic mechanisms have the ability of being vital to many regions of cancer diagnosis and therapy.

It seems that, studies that aid in understanding of FAP on a molecular level have produced significant tools for genetic testing for high-risk hereditary of the disease, predictive markers. From the evidence, we can infer that many genes (whether tumor suppressor or oncogenes) do have an impact on inherited diseases such as FAP.

Also, they could have significant roles on many pathways that associated with the disease (19, 127, 128). As an effective high-throughput tool, NGS would possibly replace current screening strategies for polyposis. Last but not least, future studies will help to describe whether special epigenetic perspectives would be necessary and allow transformation by oncogenes such as *RAS* family (124).

FAP is one of the best defined hereditary cancers that account less than 1% of all CRC incidences. Small abnormalities (develop hundreds to thousands of colorectal polyps) at the surface of the intestinal tract observed, especially in the large intestine including the colon or rectum. Untreated polyposis leads to 100% risk of cancer. If malignancy develops, this may present with weight loss. FAP can also give few or no signs until it have developed into advanced CRC (**Adapted from **https://familyhistorybowelcancer.wordpress.com/tag/familial-adenomatous-polyposis/).

CIN could occur at any stage of tumor genesis. It seems that the loss of *APC* function plays an important role in the CIN seen in FAP. The CIN pathway results from mutations in genes that control mitosis, DNA repair, and other essential processes of DNA replication. CIN is associated with mutation in *APC* gene and/or lack of chromosome 5q that includes the *APC* gene, mutation of the *KRAS* oncogene, deletion of chromosome 17p and loss of chromosome 18q, which contains the important tumor suppressor gene *TP53*. 

Within the sight of the Wnt, its receptor, Frizzled, in complex with LRP6, is actuated. *Axin *(Axil/conductin) binds to a complex with *APC, *β-Catenin, and *GSK3β* to advance β-Catenin phosphorylation**.** *GSK3β* controls this process via phosphorylating β-Catenin*, APC and Axin* (Axil/conductin) complex. Activation of the Wnt signaling pathway prevents *GSK3β* function and stabilizes β-Catenin. Loss of the negative Wnt pathway regulator *APC* takes place in the majority of colorectal cancers such as FAP. In the absence of Wnt-signaling, *GSK3β* and CK1 or 2 kinases become phosphorylate and active β-Catenin at serine and threonine residues within the N-terminal area. Mutations of β-Catenin or truncation of *APC*, which occur in FAP, increases the transcriptional activity and stability of β-Catenin. Disruption of the *APC/*β-Catenin pathway is proven as a crucial step in the improvement of FAP. 

**Table 1:** Tumor-suppressor genes commonly associated with FAP

**Gene**	**Full Name**	**Location**	**Comment**
*APC*	adenomatous polyposis coli	5q21-q22	Familial adenomas polyp, an autosomal dominant pre-malignant disease that usually progresses to malignancy is caused by mutations in the *APC* TSG. This gene counteracts with β-Catenin activation at Wnt target genes (Frequency:85).
*Axin2*	axin 2	17q24.1	*Axin2* likely plays a significant role in the regulation of the stability of β-Catenin in the Wnt pathway; it forms a complex of *APC*, β-Catenin, glycogen synthase kinase 3-beta (GSK3b), and conduction, which leads to the degradation of β-Catenin. This gene appears to act as a tumor suppressor, and somatic mutations have been seen in many different tumor types such as FAP tumors.
*DKK1*	dickkopf WNT signaling pathway inhibitor 1	10q11.2	This gene encodes a protein which is a member of the dickkopf group. *DKK1* Is a secreted protein with two cysteines rich regions and is involved in embryonic improvement via its prevention of the Wnt signaling pathway.
*TP53*	tumor protein p53	17p13.1	This gene encodes a tumor suppressor protein, including transcriptional activation, oligomerization areas and DNA binding, The encoded protein responds to varying cellular stresses to control expression of target genes, therefore inducing DNA repair, senescence, apoptosis, cell cycle arrest, or alterations in metabolism. Mutations in *TP53* are associated with a diversity of human syndrome, such as FAP. *P53* expression was only slightly increased in adenomas, but more often expressed in carcinomas in FAP patients (Frequency: 35-55).

**Table 2:** Different types of mutation in *APC* in FAP patients from different populations

**Mutation**	**Frequency (%)**	**Exon**	**Population**	**Reference**
point mutations	80	--------	Belgian	(39)
micro deletions	60-70	-------	Balearic People	(47)
Frame shift	43	15	Rochester(Mayo clinic)	(52)
nonsense	42	15	Rochester(Mayo clinic)	(52)
Duplication	------	8,14,15	Brazilian	(58)
Duplication	6	15	Rochester(Mayo clinic)	(52)
large deletions	37.5	15	Hungary	(54)
large deletions	15	14	Belgian	(39)
large deletions	14.2	11,15	Chinese	(55)
large deletions	12.5	11-13	Swedish	(53)
large deletions	7-12	14	London	(56)
large deletions	7.3	4,1-15	Spanish	(51)
large deletions	7.57	14	Taiwanese	(129)
large deletions	6	14	Czech	(57)
large deletions	5.8	15	Iranian	(50)
large deletions	5.6	3,4,10-14,11-13,14-15	Polish	(40)
large deletion	10-15	15	Balearic People	(47)
large deletion	0	--------	Greek	(46)
large deletion	0	--------	Slovak	(57)

* *APC*=adenomatous polyposis coli, FAP= familial adenomatous polyposis

**Table 3:** Wnt/β-Catenin Target Genes in FAP

**Gene**	**Comment**	**Ref**
*APC*	the most commonly mutated gene in Wnt pathway in FAP	(15, 65)
*AXIN2*	The *AXIN2* plays an important role in the adjustment of the stability of β-Catenin in the Wnt pathway	(81)
*TCF7L2*	This gene is a key effector for Wnt pathway, and the bipartite transcription factor TCF / β-Catenin, is provided by free β-Catenin and a TCF protein, including TCF7L2	(35, 64, 77-79)
*NKD1*	regulate the Wnt signaling pathway	(84, 85)
*SOX9*	It is essential for cell differentiation within the intestinal stem cell. This gene is transcriptionally repressed by Wnt signaling	(83)
*EphB2*	The *EphB2* encodes the Eph receptor B, that reported to be a target of the Wnt signaling pathway	(65, 80)

## References

[1] Haggar FA, Boushey RP (2009). Colorectal cancer epidemiology: incidence, mortality, survival, and risk factors. Clin Colon Rectal Surg.

[2] Patel SG, Ahnen DJ (2012). Familial colon cancer syndromes: an update of a rapidly evolving field. Curr Gastroenterol Rep.

[3] Chan AT, Giovannucci EL (2010). Primary prevention of colorectal cancer. Gastroenterology.

[4] Mosby TT, Cosgrove M, Sarkardei S, Platt KL, Kaina B (2012). Nutrition in adult and childhood cancer: role of carcinogens and anti-carcinogens. Anticancer Res.

[5] Tatishchev SF, VanBeek C, Wang HL (2012). Helicobacter pylori infection and colorectal carcinoma: is there a causal association?. J Gastrointest Oncol.

[6] Jasperson KW, Burt RW (2014). APC-associated Polyposis Conditions.

[7] Rengifo-Cam W, Jasperson KW, Burt RW, Samadder NJ, Boardman, LA (2016). Familial adenomatous polyposis. Intestinal Polyposis Syndromes.

[8] Waller A, Findeis S, Lee MJ (2016). Familial Adenomatous Polyposis. J Pediatr Genet.

[9] Galiatsatos P, Foulkes WD (2006). Familial adenomatous polyposis. Am J Gastroenterol.

[10] Plawski A, Banasiewicz T, Borun P, Kubaszewski L, Krokowicz P, Skrzypczak-Zielinska M (2013). Familial adenomatous polyposis of the colon. Hered Cancer Clin Pract.

[11] Kanthan R, Senger JL, Kanthan SC (2012). Molecular events in primary and metastatic colorectal carcinoma: a review. Patholog Res Int.

[12] Aoki K, Taketo MM (2007). Adenomatous polyposis coli (APC): a multi-functional tumor suppressor gene. J Cell Sci.

[13] Rivlin N, Brosh R, Oren M, Rotter V (2011). Mutations in the p53 tumor suppressor gene important milestones at the various steps of tumorigenesis. Genes Cancer.

[14] Sameer ASS (2013). Colorectal cancer: molecular mutations and polymorphisms. Front Oncol.

[15] Armaghany T, Wilson JD, Chu Q, Mills G (2012). Gastrointest Cancer Res.

[16] Bunz F (2016). Principles of Cancer Genetics.

[17] Pino MS, Chung DC (2010). The chromosomal instability pathway in colon cancer. Gastroenterology.

[18] Goss KH, Groden J (2000). Biology of the adenomatous polyposis coli tumor suppressor. J Clin Oncol.

[19] Jasperson KW, Tuohy TM, Neklason DW, Burt RW (2010). Hereditary and familial colon cancer. Gastroenterology.

[20] Migliore L, Migheli F, Spisni R, Coppedè F (2011). Genetics, cytogenetics, and epigenetics of colorectal cancer. J Biomed Biotechnol.

[21] Hadziavdić V, Eminović I, Ascerić M, Komel R (2008). Familial adenomatous polyposis: analysis of genetic instability of microsatellites Loci and genetic alternations of tumor suppressor genes. Bosn J Basic Med Sci.

[22] Markowitz SD, Bertagnolli MM (2009). Molecular basis of colorectal cancer. N Engl J Med.

[23] Fukuyama R, Niculaita R, Ng KP, Obusez E, Sanchez J, Kalady M (2008). Mutated in colorectal cancer, a putative tumor suppressor for serrated colorectal cancer, selectively represses β-catenin-dependent transcription. Oncogene.

[24] Wang J, El-Masry N, Talbot I, Tomlinson I, Alison MR, El-Bahrawy M (2013). Expression profiling of proliferation and apoptotic markers along the Adenoma-Carcinoma sequence in familial adenomatous polyposis patients. Gastroenterol Res Pract.

[25] Kashfi SMH, Behboudi Farahbakhsh F, Golmohammadi M, Nazemalhosseini Mojarad E, Azimzadeh P, Asadzadeh Aghdaie H (2014). Frameshift mutations (deletion at codon 1309 and codon 849) in the APC gene in Iranian FAP patients: a case series and review of the literature. Int J Mol Cell Med.

[26] Worthley DL, Leggett BA (2010). Colorectal cancer: molecular features and clinical opportunities. Clin Biochem Rev.

[27] Valle L (2014). Genetic predisposition to colorectal cancer: where we stand and future perspectives. World J Gastroenterol.

[28] Raskov H, Pommergaard HC, Burcharth J, Rosenberg J (2014). Colorectal carcinogenesis-update and perspectives. World J Gastroenterol.

[29] Kennedy RD, Potter DD, Moir CR, El-Youssef M (2014). The natural history of familial adenomatous polyposis syndrome: A 24year review of a single center experience in screening, diagnosis, and outcomes. J Pediatr Surg.

[30] Bisgaard ML, Bülow S (2006). Familial adenomatous polyposis (FAP): genotype correlation to FAP phenotype with osteomas and sebaceous cysts. Am J Med Genet A.

[31] Mishra N, Hall J (2012). Identification of patients at risk for hereditary colorectal cancer. Clin Colon Rectal Surg.

[32] Kwong LN, Dove WF (2009). APC and its modifiers in colon cancer. Adv Exp Med Biol.

[33] Fearon ER (2011). Molecular genetics of colorectal cancer. Annu Rev Pathol.

[34] Half E, Bercovich D, Rozen P (2009). Familial adenomatous polyposis. Orphanet J Rare Dis.

[35] Fearnhead NS, Britton MP, Bodmer WF (2001). The ABC of APC. Hum Mol Gen.

[36] Han SH, Ryu JS, Kim YJ, Cho HI, Yang YH, Lee KR (2011). Mutation analysis of the APC gene in unrelated Korean patients with FAP: four novel mutations with unusual phenotype. Fam Cancer.

[37] Heinen CD (2010). Genotype to phenotype: analyzing the effects of inherited mutations in colorectal cancer families. Mutat Res.

[38] Martayan A, Sanchez-Mete L, Baldelli R, Falvo E, Barnabei A, Conti L (2010). Gene variants associated to malignant thyroid disease in familial adenomatous polyposis: a novel APC germline mutation. J Endocrinol Invest.

[39] Michils G, Tejpar S, Thoelen R, Cutsem Ev, Vermeesch JR, Fryns JP (2005). Large deletions of the APC gene in 15% of mutation‐negative patients with classical polyposis (FAP): A Belgian study. Hum Mutat.

[40] Plawski A, Slomski R (2008). APC gene mutations causing familial adenomatous polyposis in Polish patients. J Appl Genet.

[41] Gavert N, Yaron Y, Naiman T, Bercovich D, Rozen P, Shomrat R (2002). Molecular analysis of the APC gene in 71 Israeli families: 17 novel mutations. Hum Mutat.

[42] Rosa MD, Scarano MI, Panariello L, Morelli G, Riegler G, Rossi GB (2003). The mutation spectrum of the APC gene in FAP patients from southern Italy: detection of known and four novel mutations. Hum Mutat.

[43] Bertario L, Russo A, Sala P, Varesco L, Giarola M, Mondini P (2003). Multiple approach to the exploration of genotype-phenotype correlations in familial adenomatous polyposis. J Clin Oncol.

[44] Mihalatos M, Apessos A, Dauwerse H, Velissariou V, Psychias A, Koliopanos A (2005). Rare mutations predisposing to familial adenomatous polyposis in Greek FAP patients. BMC Cancer.

[45] Su LK, Kohlmann W, Ward PA, Lynch PM (2002). Different familial adenomatous polyposis phenotypes resulting from deletions of the entire APC exon 15. J Hum Genet.

[46] Fostira F, Thodi G, Sandaltzopoulos R, Fountzilas G, Yannoukakos D (2010). Mutational spectrum of APC and genotype-phenotype correlations in Greek FAP patients. BMC Cancer.

[47] González S, Blanco I, Campos O, Julià M, Reyes J, Llompart A (2005). Founder mutation in familial adenomatous polyposis (FAP) in the Balearic Islands. Cancer Genet Cytogenet.

[48] Moisio AL, Järvinen H, Peltomäki P (2002). Genetic and clinical characterisation of familial adenomatous polyposis: a population based study. Gut.

[49] Farahani RK, Haghighi MM, Aghdaei HA, Keshavarzi F, Taleghani M, Zali MR (2014). Adenomatous polyposis coli gene large deletions in Iranian patients with familial adenomatous polyposis. Indian J Cancer.

[50] Aghdaei HA (2015). Novel missense mutation at codon 2774 (C 8321 G> A) p S2774N of APC gene in a Denovo case of familial adenomatous polyposis. Arch Iran Med.

[51] Gómez-Fernández N, Castellví-Bel S, Fernández-Rozadilla C, Balaguer F, Muñoz J, Madrigal I (2009). Molecular analysis of the APC and MUTYH genes in Galician and Catalonian FAP families: a different spectrum of mutations. BMC Med Genet.

[52] Kerr SE, Thomas CB, Thibodeau SN, Ferber MJ, Halling KC (2013). APC germline mutations in individuals being evaluated for familial adenomatous polyposis: a review of the Mayo Clinic experience with 1591 consecutive tests. J Mol Diagn.

[53] Meuller J, Kanter-Smoler G, Nygren AO, Errami A, Grönberg H, Holmberg E (2004). Identification of genomic deletions of the APC gene in familial adenomatous polyposis by two independent quantitative techniques. Genet Test.

[54] Papp J, Kovacs ME, Matrai Z, Orosz E, Kásler M, Børresen-Dale AL (2016). Contribution of APC and MUTYH mutations to familial adenomatous polyposis susceptibility in Hungary. Fam Cancer.

[55] Sheng JQ, Cui WJ, Fu L, Jin P, Han Y, Li SJ (2010). APC gene mutations in Chinese familial adenomatous polyposis patients. World J Gastroenterol.

[56] Sieber O, Lamlum H, Crabtree M, Rowan A, Barclay E, Lipton L (2002). Whole-gene APC deletions cause classical familial adenomatous polyposis, but not attenuated polyposis or “multiple” colorectal adenomas. Proc Natl Acad Sci USA.

[57] Stekrova J, Sulova M, Kebrdlova V, Zidkova K, Kotlas J, Ilencikova D (2007). Novel APC mutations in Czech and Slovak FAP families: clinical and genetic aspects. BMC Med Genet.

[58] Torrezan GT, da Silva FCC, Santos ÉMM, Krepischi ACV, Achatz MIW, Junior SA (2013). Mutational spectrum of the APC and MUTYH genes and genotype-phenotype correlations in Brazilian FAP, AFAP, and MAP patients. Orphanet J Rare Dis.

[59] Lipton L, Tomlinson I (2006). The genetics of FAP and FAP-like syndromes. Fam Cancer.

[60] Ruiz‐Ponte C, Vega A, Carracedo A, Barros F (2001). Mutation analysis of the adenomatous polyposis coli (APC) gene in northwest Spanish patients with familial adenomatous polyposis (FAP) and sporadic colorectal cancer. Hum Mutat.

[61] Schlussel AT, Donlon SS, Eggerding FA, Gagliano RA (2014). Identification of an APC variant in a patient with clinical attenuated familial adenomatous polyposis. Case Rep Med.

[62] Sierra J, Yoshida T, Joazeiro CA, Jones KA (2006). The APC tumor suppressor counteracts β-catenin activation and H3K4 methylation at Wnt target genes. Genes Dev.

[63] Syngal S, Brand RE, Church JM, Giardiello FM, Hampel HL, Burt RW (2015). ACG clinical guideline: Genetic testing and management of hereditary gastrointestinal cancer syndromes. Am J Gastroenterol.

[64] Najdi R, Holcombe RF, Waterman ML (2011). Wnt signaling and colon carcinogenesis: beyond APC. J Carcinog.

[65] Schneikert J, Behrens J (2007). The canonical Wnt signalling pathway and its APC partner in colon cancer development. Gut.

[66] Rao CV, Yamada HY (2013). Genomic instability and colon carcinogenesis: from the perspective of genes. Front Oncol.

[67] Alberici P, de Pater E, Cardoso J, Bevelander M, Molenaar L, Jonkers J (2007). Aneuploidy arises at early stages of APC-driven intestinal tumorigenesis and pinpoints conserved chromosomal loci of allelic imbalance between mouse and human. Am J Pathol.

[68] Arvelo F, Sojo F, Cotte C (2015). Biology of colorectal cancer. Ecancermedicalscience.

[69] Safaei A, Sobhi S, Rezaei-Tavirani M, Zali MR (2013). Genomic and epigenetic instability in colorectal cancer. Iran J Cancer Prev.

[70] Dalton WB, Yang VW (2007). Mitotic origins of chromosomal instability in colorectal cancer. Current Colorectal Cancer Rep.

[71] Hagan S, Orr MC, Doyle B (2013). Targeted therapies in colorectal cancer:an integrative view by PPPM. EPMA J.

[72] Wang H, Liang L, Fang J, Xu J (2016). Somatic gene copy number alterations in colorectal cancer: New quest for cancer drivers and biomarkers. Oncogene.

[73] Das S, Yu S, Sakamori R, Stypulkowski E, Gao N (2012). Wntless in Wnt secretion: molecular, cellular and genetic aspects. Front Biol.

[74] MacDonald BT, Tamai K, He X (2009). Wnt/β-catenin signaling: components, mechanisms, and diseases. Developmental Cell.

[75] Narayan S, Roy D (2003). Role of APC and DNA mismatch repair genes in the development of colorectal cancers. Mol Cancer.

[76] Cancer Genome Atlas Network (2012). Comprehensive molecular characterization of human colon and rectal cancer. Nature.

[77] Onyido EK, Sweeney E, Nateri AS (2016). Wnt-signalling pathways and microRNAs network in carcinogenesis: experimental and bioinformatics approaches. Mol Cancer.

[78] Cadigan KM, Waterman ML (2012). TCF/LEFs and Wnt signaling in the nucleus. Cold Spring Harb Perspect Biol.

[79] Bush BM, Brock AT, Deng JA, Nelson RA, Sumter TF (2013). The Wnt/β‐catenin/T‐cell factor 4 pathway up‐regulates high‐mobility group A1 expression in colon cancer. Cell Biochem Funct.

[80] Bowden NA, Croft A, Scott RJ (2007). Gene expression profiling in familial adenomatous polyposis adenomas and desmoid disease. Hered Cancer Clin Pract.

[81] Mazzoni SM, Fearon ER (2014). AXIN1 and AXIN2 variants in gastrointestinal cancers. Cancer Letters.

[82] Benchabane H, Ahmed Y (2009). The adenomatous polyposis coli tumor suppressor and Wnt signaling in the regulation of apoptosis. Adv Exp Med Biol.

[83] Blache P, van de Wetering M, Duluc I, Domon C, Berta P, Freund JN (2004). SOX9 is an intestine crypt transcription factor, is regulated by the Wnt pathway, and represses the CDX2 and MUC2 genes. J Cell Biol.

[84] Caldwell G, Jones C, Ashley A, Wei W, Hejmadi R, Morton D (2010). Wnt signalling in adenomas of familial adenomatous polyposis patients. Br J Cancer.

[85] Stancikova J, Krausova M, Kolar M, Fafilek B, Svec J, Sedlacek R (2015). NKD1 marks intestinal and liver tumors linked to aberrant Wnt signaling. Cell Signal.

[86] Tezcan G, Tunca B, Ak S, Cecener G, Egeli U (2016). Molecular approach to genetic and epigenetic pathogenesis of early-onset colorectal cancer. World J Gastrointest Oncol.

[87] Schnekenburger M, Diederich M (2012). Epigenetics offer new horizons for colorectal cancer prevention. Curr Colorectal Cancer Rep.

[88] Mojarad EN, Kuppen PJ, Aghdaei HA, Zali MR (2013). The CpG island methylator phenotype (CIMP) in colorectal cancer. Gastroenterol Hepatol Bed Bench.

[89] Vavouri T, Lehner B (2012). Human genes with CpG island promoters have a distinct transcription-associated chromatin organization. Genome Biol.

[90] Bardhan K, Liu K (2013). Epigenetics and colorectal cancer pathogenesis. Cancers.

[91] Wynter CV, Kambara T, Walsh MD, Leggett BA, Young J, Jass JR (2006). DNA methylation patterns in adenomas from FAP, multiple adenoma and sporadic colorectal carcinoma patients. ‎Int J Cancer.

[92] Brait M, Ling S, Nagpal JK, Chang X, Park HL, Lee J (2012). Cysteine dioxygenase 1 is a tumor suppressor gene silenced by promoter methylation in multiple human cancers. PLoS One.

[93] Kazanets A, Shorstova T, Hilmi K, Marques M, Witcher M (2016). Epigenetic silencing of tumor suppressor genes: Paradigms, puzzles, and potential. Biochim Biophys Acta.

[94] Zhang Y, Chen S, Zhu M, Li J, Ma G, Zhang X (2008). Promoter hypermethylation and loss of heterozygosity of the APC gene in patients with familial adenomatous polyposis [in Chinese]. Zhonghua Yi Xue Yi Chuan Xue Za Zhi.

[95] Segditsas S, Sieber OM, Rowan A, Setien F, Neale K, Phillips RK (2008). Promoter hypermethylation leads to decreased APC mRNA expression in familial polyposis and sporadic colorectal tumours, but does not substitute for truncating mutations. Exp Mol Pathol.

[96] Pavicic W, Nieminen TT, Gylling A, Pursiheimo JP, Laiho A, Gyenesei A (2014). Promoter‐specific alterations of APC are a rare cause for mutation‐negative familial adenomatous polyposis. Genes Chromosomes and Cancer.

[97] Bannister AJ, Kouzarides T (2011). Regulation of chromatin by histone modifications. Cell Research.

[98] Lao VV, Grady WM (2011). Epigenetics and colorectal cancer. Nat Rev Gastroenterol Hepatol.

[99] Sadikovic B, Al-Romaih K, Squire J, Zielenska M (2008). Cause and consequences of genetic and epigenetic alterations in human cancer. Curr Genomics.

[100] Hajieghrari B, Farrokhi N, Goliaei B, Kavousi K (2015). Computational Identification, Characterization and Analysis of Conserved miRNAs and their Targets in Amborella Trichopoda. J Data Mining Genomics Proteomics.

[101] Hajieghrari B, Farrokhi N, Goliaei B, Kavousi K (2016). Identification and Characterization of Novel miRNAs in Chlamydomonas reinhardtii by Computational Methods. Microrna.

[102] Wu WK, Law PT, Lee CW, Cho CH, Fan D, Wu K (2011). MicroRNA in colorectal cancer: from benchtop to bedside. Carcinogenesis.

[103] Manikandan J, Aarthi JJ, Kumar SD, Pushparaj PN (2008). Oncomirs: the potential role of non-coding microRNAs in understanding cancer. Bioinformation.

[104] Hummel R, Hussey DJ, Haier J (2010). MicroRNAs: predictors and modifiers of chemo-and radiotherapy in different tumour types. Eur J Cancer.

[105] Hutchison J, Cohen Z, Onyeagucha BC, Funk J, Nelson MA (2013). How microRNAs influence both hereditary and inflammatory-mediated colon cancers. Cancer Genet.

[106] Tsuchiya N, Ochiai M, Nakashima K, Ubagai T, Sugimura T, Nakagama H (2007). SND1, a component of RNA-induced silencing complex, is up-regulated in human colon cancers and implicated in early stage colon carcinogenesis. Cancer Res.

[107] Nagel R, le Sage C, Diosdado B, van der Waal M, Vrielink JAO, Bolijn A (2008). Regulation of the adenomatous polyposis coli gene by the miR-135 family in colorectal cancer. Cancer Res.

[108] Kamatani A, Nakagawa Y, Akao Y, Maruyama N, Nagasaka M, Shibata T (2013). Downregulation of anti-oncomirs miR-143/145 cluster occurs before APC gene Aberration in the Development of Colorectal Tumors. Med Mol Morphol.

[109] Yamaguchi T, Iijima T, Wakaume R, Takahashi K, Matsumoto H, Nakano D (2014). Underexpression of miR-126 and miR-20b in hereditary and nonhereditary colorectal tumors. Oncology.

[110] Chen WS, Leung CM, Pan HW, Hu LY, Li SC, Ho MR (2012). Silencing of miR-1-1 and miR-133a-2 cluster expression by DNA hypermethylation in Colorectal Cancer. Oncol Rep.

[111] Stuppia L, Antonucci I, Palka G, Gatta V (2012). Use of the MLPA assay in the molecular diagnosis of gene copy number alterations in human genetic diseases. Int J Mol Sci.

[112] Macdonald F, Payne SJ (2011). Best practice guidelines for molecular analysis of colorectal polyposis: familial adenomatous polyposis coli (FAP) and MUTYH-associated polyposis (MAP).

[113] Fassan M, Simbolo M, Bria E, Mafficini A, Pilotto S, Capelli P (2014). High-throughput mutation profiling identifies novel molecular dysregulation in high-grade intraepithelial neoplasia and early gastric cancers. Gastric Cancer.

[114] Shendure J, Ji H (2008). Next-generation DNA sequencing. Nat Biotechnol.

[115] Kahvejian A, Quackenbush J, Thompson JF (2008). What would you do if you could sequence everything?. Nat Biotechnol.

[116] Simbolo M, Mafficini A, Agostini M, Pedrazzani C, Bedin C, Urso ED (2015). Next-generation sequencing for genetic testing of familial Colorectal Cancer Syndromes. Hered Cancer Clin Pract.

[117] Hegde M, Ferber M, Mao R, Samowitz W, Ganguly A (2013). ACMG technical standards and guidelines for genetic testing for inherited colorectal cancer (Lynch syndrome, familial adenomatous polyposis, and MYH-associated polyposis). Genet Med.

[118] Robanus-Maandag E, Bosch C, Amini-Nik S, Knijnenburg J, Szuhai K, Cervera P (2011). Familial adenomatous polyposis-associated desmoids display significantly more genetic changes than sporadic desmoids. PLoS One.

[119] Leoz ML, Carballal S, Moreira L, Ocaña T, Balaguer F (2015). The genetic basis of familial adenomatous polyposis and its implications for clinical practice and risk management. Appl Clin Genet.

[120] MacDonald BT, He X (2012). Frizzled and LRP5/6 receptors for Wnt/β-catenin signaling. Cold Spring Harb Perspect Biol.

[121] Phelps RA, Broadbent TJ, Stafforini DM, Jones DA (2009). New perspectives on APC control of cell fate and proliferation in colorectal cancer. Cell Cycle.

[122] Phelps RA, Chidester S, Dehghanizadeh S, Phelps J, Sandoval IT, Rai K (2009). A two-step model for colon adenoma initiation and progression caused by APC loss. Cell.

[123] Amos-Landgraf JM, Kwong LN, Kendziorski CM, Reichelderfer M, Torrealba J, Weichert J (2007). A target-selected Apc-mutant rat kindred enhances the Modeling of Familial Human Colon Cancer. Proc Natl Acad Sci USA.

[124] Hammoud SS, Cairns BR, Jones DA (2013). Epigenetic regulation of Colon Cancer and intestinal stem cells. Curr Opin Cell Biol.

[125] Kadiyska T, Todorov T, Bichev S, Vazharova R, Nossikoff A, Savov A (2014). APC promoter 1B deletion in familial polyposis:implications for mutation‐negative families. Clin Genet.

[126] Kashfi SMH, Golmohammadi M, Behboudi F, Nazemalhosseini-Mojarad E, Zali MR (2013). MUTYH the base excision repair gene family member associated with colorectal cancer polyposis. Gastroenterol Hepatol Bed Bench.

[127] Pritchard CC, Grady WM (2010). Colorectal cancer molecular biology moves into clinical practice. Gut.

[128] Reimers MS, Zeestraten EC, Kuppen PJ, Liefers GJ, van de Velde CJ (2013). Biomarkers in precision therapy in colorectal cancer. Gastroenterol Rep.

[129] Chiang JM, Chen H, Tang R, Chen JS, Changchien C, Hsieh P (2010). Mutation analysis of the APC gene in Taiwanese FAP families: low incidence of APC germline mutation in a distinct subgroup of FAP families. Fam Cancer.

